# Cellular Senescence in Neurodegeneration: From Cell Types to Therapeutic Opportunities

**DOI:** 10.3390/biomedicines14040758

**Published:** 2026-03-26

**Authors:** Marta Zawadzka, Julia Rydzek, Julia Lizon, Zuzanna Krupa, Joanna Wrona, Sławomir Woźniak

**Affiliations:** 1 Student Scientific Society Anatomia-Klinika-Nauka, Division of Anatomy, Department of Human Morphology and Embryology, Wroclaw Medical University, 50-367 Wroclaw, Poland; julia.rydzek@student.umw.edu.pl (J.R.); julia.lizon@student.umw.edu.pl (J.L.); zuzanna.krupa@student.umw.edu.pl (Z.K.); joanna.wrona@student.umw.edu.pl (J.W.); 2 Division of Anatomy, Department of Human Morphology and Embryology, Wroclaw Medical University, 50-367 Wroclaw, Poland; slawomir.wozniak@umw.edu.pl

**Keywords:** cellular senescence, neurodegenerative diseases, neuroinflammation, senescence-associated secretory phenotype (SASP), central nervous system aging

## Abstract

Neurodegenerative diseases of the central nervous system, such as Alzheimer’s disease, Parkinson’s disease, and multiple sclerosis, represent a growing health challenge in ageing populations. Among the mechanisms underlying these disorders, increasing attention has been directed toward the role of cellular senescence. This process, triggered by chronic cellular and oxidative stress as well as DNA damage, leads to irreversible cell-cycle arrest and the development of the senescence-associated secretory phenotype (SASP). Within the central nervous system, the accumulation of senescent cells induces chronic inflammation, blood–brain barrier disruption, and progression of neurodegenerative processes. In this review, we present current evidence regarding the mechanisms of cellular senescence in the central nervous system, with particular emphasis on the role of SASP in neuroinflammation, vascular dysfunction, and neural tissue damage. Experimental and clinical data supporting the involvement of cellular senescence in the pathogenesis of Alzheimer’s disease, Parkinson’s disease, and multiple sclerosis are discussed. The review also covers methods for identifying senescent cells in the brain, including molecular marker-based approaches and machine learning-based tools. Importantly, we discuss the methodological limitations of commonly used senescence markers, such as their limited specificity and the risk of false-positive detection, particularly in the heterogeneous cellular environment of the central nervous system. Strategies to improve detection reliability discussed in this review include the use of multimarker signatures, analysis of SASP components using qRT-PCR and ELISA, as well as transcriptomic approaches such as RNA sequencing and single-cell RNA sequencing. Furthermore, we analyze therapeutic strategies targeting senescent cells—senolytics, senomorphics, and SASP modulation—together with their limitations and associated clinical challenges. The collected evidence indicates that precise characterization of senescent cell populations in the brain is essential for the development of disease-modifying therapies for neurodegenerative disorders.

## 1. Introduction

Neurodegenerative diseases are a heterogeneous group characterized by the progressive and irreversible loss of neuronal structure or function, often accompanied by the accumulation of misfolded proteins, synaptic dysfunction, and neuronal death [1,2,3]. Many mechanisms contribute to progressive neuronal loss, and one of great importance for the development of neurodegenerative diseases is cellular senescence. Senescent cells accumulate in the central nervous system and may influence the pathogenesis of various neurodegenerative disorders, such as Alzheimer’s disease (AD), Parkinson’s disease (PD), amyotrophic lateral sclerosis, multiple sclerosis (MS) or Huntington’s disease [4]. These conditions typically manifest as gradually worsening impairments in cognition, motor control, behavior, or autonomic function. Neurodegenerative diseases represent a growing global health concern, driven largely by increased life expectancy and the progressive aging of populations; therefore, they pose a major challenge for public health in developed countries [5,6]. Despite significant research efforts, the mechanisms driving progressive neuronal dysfunction and cell loss remain not fully elucidated.

Cellular senescence is a physiological process that occurs in the human body and represents a state of irreversible cell-cycle arrest that arises in response to telomere erosion, DNA damage, oncogene activation, oxidative stress, and other cellular stressors [7,8,9]. Senescent cells are characterized by the induction of p16, p21, p-RB, and SA-β-gal, accompanied by broad changes in gene expression, chromatin organization, and cellular metabolism. A hallmark of senescence is the development of the senescence-associated secretory phenotype (SASP) [4].

The SASP is a pro-inflammatory secretory program that triggers the release of a broad range of cytokines, chemokines, and related inflammatory mediators that can influence surrounding cells and tissues, for example by activating microglial responses in the brain [10]. Although senescence contributes to normal processes such as tissue development, wound healing, and tumor suppression, the accumulation of senescent cells with age promotes chronic inflammation, tissue dysfunction, and increased vulnerability to age-related diseases, including neurodegenerative diseases [9,10].

Not only mitotically competent cells, including astrocytes, microglia, oligodendrocytes, and endothelial cells, can undergo senescence, but post-mitotic neurons may also develop senescent features [8]. Senescent cells accumulate with age and secrete SASP factors, inducing a strong inflammatory response in the CNS, damaging neural tissue, and promoting the deposition of pathological proteins [9,10].

The accumulation of senescent cells is observed in neurodegenerative diseases such as AD, PD, MS and others [4]. In AD, senescent neurons, microglia, and astrocytes contribute to enhanced β-amyloid and tau deposition [4]. In PD, senescence of dopaminergic neurons and microglia coincides with Lewy body formation and neuroinflammation [11]. In MS, cells exhibiting a senescent phenotype have likewise been identified [12].

Continued research efforts are required to advance our understanding of how widespread the effects of cellular senescence are in neurodegenerative disorders and to translate these insights into effective therapeutic strategies [4,10]. Although studies have linked senescence-related alterations to neurodegenerative pathology, a comprehensive integration of cell-type-specific mechanisms and therapeutic implications remains lacking. Advances in technology enable the identification of senescent subtypes present at sites of age-related brain disorders. This is of considerable importance, as anti-senescence approaches require the selective targeting of specific senescent cell populations. Consequently, precise characterization of cellular senescence subtypes is essential for the development of effective interventions for neurodegenerative disorders [4,9].

In this review, we focus on synthesizing current knowledge on cellular senescence in the nervous system and examining its contribution to major neurodegenerative diseases. We also highlight the major challenges associated with emerging therapeutic strategies that target senescent cells and their secretory profile.

## 2. Materials and Methods

This review was conducted as a structured narrative literature review aimed at synthesizing current evidence regarding the role of cellular senescence in neurodegenerative diseases of CNS. The methodology followed general principles of transparent literature synthesis recommended for narrative reviews, including a predefined search strategy, explicit inclusion criteria, and a multistep screening process.

### 2.1. Literature Search Strategy

A comprehensive literature search was performed in three major biomedical databases: PubMed, Scopus, and Web of Science. The search covered publications from January 2005 to June 2025, reflecting the period during which research on cellular senescence SASP in neurodegeneration has significantly expanded. Search queries combined controlled vocabulary and free-text terms using Boolean operators (AND, OR). The main keywords included: “cellular senescence”, “senescence-associated secretory phenotype” OR “SASP”, “neurodegeneration” OR “neurodegenerative diseases”, “Alzheimer’s disease”, “Parkinson’s disease”, “multiple sclerosis”, “neuroinflammation”, “astrocytes”, “microglia”, “blood–brain barrier”, “senolytics”, “senomorphics”. These terms were combined in various configurations to capture studies addressing both mechanistic and therapeutic aspects of cellular senescence in CNS disorders. Reference lists of key articles and recent reviews were also manually screened to identify additional relevant studies.

### 2.2. Study Selection Process

The literature selection process consisted of three stages:

1. Identification—all records retrieved from database searches were exported and duplicate publications were removed.
2. Screening—titles and abstracts were independently screened by the authors to assess relevance to the topic of cellular senescence in neurodegeneration.
3. Eligibility assessment—potentially relevant studies underwent full-text evaluation.

Discrepancies in study selection were resolved through discussion among the authors. Although this review was not conducted as a formal systematic review, the study identification and screening procedures were designed to improve transparency and reduce selection bias.

### 2.3. Inclusion and Exclusion Criteria

Studies were included if they met the following criteria:

1. Investigated cellular senescence or SASP in the central nervous system;
2. Addressed mechanisms linking senescence to neurodegenerative diseases;
3. Examined senescence-related changes in CNS cell populations, including neurons, astrocytes, microglia, oligodendrocyte lineage cells, or vascular cells;
4. Reported experimental, translational, or clinical evidence;
5. Were published in peer-reviewed journals;
6. Were available in English.

Studies were excluded if they:

1. Focused exclusively on non-neurological diseases;
2. Were conference abstracts, editorials, or commentaries;
3. Did not provide mechanistic or disease-relevant insights related to neurodegeneration.

### 2.4. Assessment of Methodological Relevance

Given the narrative nature of the review and the heterogeneity of available evidence (including experimental models, animal studies, and clinical research), a formal quantitative risk-of-bias assessment was not performed. However, methodological rigor was considered during study selection.

Priority was given to: original experimental and translational studies, human tissue studies and clinical investigations, high-quality mechanistic studies published in peer-reviewed journals, recent systematic reviews providing conceptual context.

This approach allowed the review to integrate findings from diverse experimental systems while maintaining a focus on methodologically robust and widely cited evidence.

### 2.5. Data Synthesis

The selected literature was analyzed using a qualitative narrative synthesis. Studies were grouped thematically according to key aspects of cellular senescence in neurodegeneration, including:

1. Fundamental mechanisms of cellular senescence in CNS cells;
2. Biomarkers and detection methods;
3. Roles of SASP in neuroinflammation and neurovascular dysfunction;
4. Disease-specific evidence in AD, PD and MS;
5. Therapeutic strategies targeting senescent cells.

This thematic approach enabled integration of mechanistic insights with emerging translational and therapeutic perspectives.

## 3. Fundamentals of Cellular Senescence in the Context of the Central Nervous System

Cellular senescence is a cellular response to various stressors, including DNA damage, oxidative stress, and telomere erosion, ultimately leading to cell cycle arrest. Age-associated functional decline occurs not only at the cellular level but also at molecular, tissue, and organismal levels. Cellular senescence is known to be linked to numerous pathologies such as atherosclerosis, sarcopenia, pulmonary or renal failure, neurodegeneration, and major neurodegenerative diseases including AD and PD [13]. Senescent cells exhibit characteristic molecular and morphological features that distinguish them from other cellular populations. Their typical hallmarks include cell cycle arrest, SASP, metabolic dysregulation, and macromolecular alterations [14]. A key common feature of senescent cells is irreversible cell cycle arrest mediated by various signaling pathways, such as p53–p21^WAF1/Cip1^/p16^INK4a^–RB. Although this arrest is considered irreversible in senescent cells, re-entry into the cell cycle may occur under certain conditions, as observed in cancer cells [15]. Accordingly, some studies suggest that escape from cellular senescence is required for tumor development into overt malignancy [16]. Growth arrest of damaged cells occurs in the G1 phase in response to cellular damage via p53, a tumor suppressor and cell cycle checkpoint protein [17], and through accumulation of cyclin-dependent kinase (CDK) inhibitors p16 and p21, preventing DNA replication. p21 has also been shown to participate in cell cycle arrest in response to damage occurring in the G2 phase [18]. Changes in CDK activity may contribute to cell cycle re-entry. Reduced p53 signaling leads to heterogeneous p21 levels among cells and increased CDK2 activity, enabling re-entry into the cell cycle. Thus, p53 and p21 are not only responsible for induction of cell cycle arrest but are also essential for its long-term maintenance [19].

SASP is a hallmark phenotype of senescent cells, characterized by secretion of various factors including cytokines, chemokines (which recruit immune cells), growth factors, bradykinins, miRNAs, factors inducing stem cell dysfunction (e.g., activin A), and extracellular matrix-damaging components such as proteases [20]. Depending on cell type and stimulus, SASP factors may induce diverse physiological outcomes, ranging from beneficial effects such as tissue repair to harmful effects such as tumorigenesis [21]. SASP is regulated at the levels of transcription, translation, mRNA stability, and secretion [22]. At the transcriptional level, SASP activation requires persistent DNA damage response (DDR) signaling but is independent of classical cell cycle arrest inducers in senescent cells (p21, p16, and p53) [14]. SASP is also regulated through epigenetic mechanisms. Chronic DNA damage leads to reduced dimethylation of histone H3 lysine 9, enhancing induction of inflammatory cytokines IL-6 and IL-8 [23]. The discovery of SASP helped explain why even a small number of senescent cells within tissues can exert a disproportionately large detrimental effect on tissue function and induce systemic consequences. Chronic SASP activity may accelerate aging of healthy cells, promote fibrosis, induce inflammation, and impair progenitor cell function. Furthermore, senescent cells exhibit resistance to cell death, for example through activation of anti-apoptotic pathways, and together with the age-related decline in immune clearance capacity, this promotes their accumulation in tissues [24].

Neurons are long-lived, post-mitotic cells with specialized structures and functions. With aging, neurons undergo morphological and functional changes caused by accumulation of cellular damage products, toxins, and misfolded proteins, resulting in impaired function and increased susceptibility to disease [25]. As post-mitotic cells, neurons do not undergo classical cell cycle arrest; therefore, their aging relies on mechanisms distinct from those of proliferating cells. Many hallmark features of cellular senescence including DNA damage, elevated senescence-associated β-galactosidase (SA-β-gal) activity, SASP, and increased mitochondrial production of reactive oxygen species (ROS) are largely present in neuronal cells [15]. Although neurons do not divide and do not experience classical replicative senescence (RS), they nonetheless exhibit typical senescence-associated features. In aged neurons, DNA mutations, double-strand breaks (DSBs), and oxidative lesions (single-strand breaks and 8-oxoguanine) accumulate [25]. These DNA lesions are likely caused by exposure to high levels of free radicals, which may be related to the neurons’ high demand for oxidative phosphorylation [26]. Neurons in the aging brain and peripheral nervous system accumulate various forms of DNA damage, including DSBs and focal DNA lesions. Elevated ROS levels in aging neurons result from high metabolic activity and limited antioxidant protection, and ROS is a known DNA-damaging factor in non-pathological, age-related brain aging [27]. Morphological nuclear alterations also occur in aging neural cells. Significant loss of lamin B1, a major component of the nuclear lamina, has been identified as a marker of astrocyte senescence. Reduced lamin B1 levels were observed in the granule cell layer of the human hippocampus in post-mortem tissue from elderly individuals without dementia. Differences in lamin B1 levels and nuclear morphology between granule and polymorphic layers of the hippocampus indicate regional heterogeneity in astrocyte aging [28].

A characteristic feature of the aging brain is the accumulation of the autofluorescent pigment lipofuscin in neurons [29]. Alongside this age-related process, neuromelanin gradually accumulates in neurons rich in dopamine and noradrenaline. With age, neuromelanin accumulates in neurons rich in dopamine and noradrenaline. Neuromelanin is a macromolecular substance that naturally accumulates in catecholaminergic neurons and consists mainly of eumelanin and pheomelanin. These same neurons are among the first to degenerate in PD, supporting a link between neuromelanin and neuronal vulnerability [30]. Excess neuromelanin may damage dopaminergic neurons through microglial activation. It may also induce mitochondrial dysfunction by releasing iron, generating oxidative stress, and contributing to reduced activity of the 26S proteasome [25].

In the aging brain, upregulation of pro-inflammatory cytokines and their modulators correlates with microglial changes. Neuroinflammation is also promoted by astrocytes [25]. Senescent brain cells may further support neuroinflammation through SASP factors and damage-associated molecular patterns (DAMPs) [31]. Neurons primarily communicate via electrochemical signaling, which is essential for proper brain function. During neuronal senescence, disruption of this signaling occurs, contributing to brain dysfunction not only through abnormal electrical activity but also through secretion of neuronal senescence-associated secretory phenotype (NASP) factors together with typical SASP components [32]. In one study, long-term cultures of cortical neurons acquired senescence features, including accumulation of the transcription factor GATA4, which regulates SASP secretion. Senescent neurons primarily secreted chemokines and cytokines such as MCP-1, RANTES, MIP-2, GRO-1, MCP-3, and EOTAXIN, while IL-6 and other typical SASP components were detected at low levels. Factors secreted by senescent neurons may exert paracrine effects on neighboring cells, inducing premature senescence. However, young neurons were resistant to SASP secreted by senescent cortical neurons and did not exhibit senescence markers [33] (see Table 1).

Senescent astrocytes also acquire a SASP and secrete pro-inflammatory factors such as IL-6, IL-1β, TNF-α, IL-8, and NOS2, which exert neurotoxic effects. This paracrine activity may affect various neuronal populations, including mature neurons, motor neurons, progenitor cells, and neural stem cells. Senescent astrocytes contribute to glutamate metabolism dysregulation characteristic of neurodegenerative diseases, including tauopathies, leading to neuronal death. Inhibition of astrocyte senescence reduces SASP and protects neurons in vitro, while clearance of senescent astrocytes in vivo prevents progression of neurodegeneration and cognitive decline [34]. Microglial senescence plays a critical role in increasing susceptibility to neuronal degeneration. Senescent microglia enter irreversible cell cycle arrest and acquire a SASP, exhibiting elevated levels of pro-inflammatory cytokines such as TNF-α, IL-6, and IL-8, driven by activation of p38 kinase and nuclear factor NF-κB signaling pathways. This phenotype is observed both during physiological brain aging and in regions affected by neurodegenerative diseases, such as the substantia nigra in PD and the hippocampus in AD. Currently, SASP and elevated ferritin levels are considered widely accepted markers of senescent microglia [35].

## 4. Methods and Biomarkers for Detecting Cellular Senescence in the Brain

The characteristics of senescent cells are heterogeneous and depend on both the original cell type and the specific stressor involved in the senescence process [36]. Although multiple markers can be used to identify senescent cells, unambiguous identification requires the detection of several overlapping phenotypes within the same cell, including morphological alterations, cell cycle arrest, and the SASP [37]. Evidence of senescence in the CNS can be obtained using cell-type-specific markers: in astrocytes, these include p16, p21, SA-β-gal activity, loss of HMGB1, and SASP components; in microglia, p16 and protein marker panels; in neurons, p21 and Gdf11 knockout; and in other CNS cell types such as oligodendrocyte progenitor cells (OPCs), vascular smooth muscle cells (VSMCs), and neuroblasts [38]. These methods and biomarkers are summarized in Table 2.

The SA-β-gal assay, due to its high efficiency in detecting senescent cells, can be used as a reference or control assay for other methods; however, it is considered destructive to cells and is prone to false-positive results [39]. Identification of SASP-specific markers, distinct from generalized inflammatory responses, and the analysis of multimarker signatures—such as the co-expression of pro-inflammatory cytokines (IL-6 and IL-8) with growth regulators and extracellular matrix modulators—are particularly relevant in chronic inflammatory conditions that induce cellular senescence. Quantitative reverse transcription PCR (qRT-PCR) enables the quantitative assessment of transcriptional activation of SASP components, including chemokines, pro-inflammatory cytokines, and growth factors. Due to its high sensitivity and specificity, qRT-PCR is an important technique for studying SASP gene dynamics both in vitro and in vivo [40]. High-throughput qRT-PCR platforms further facilitate comparative analyses by allowing the simultaneous measurement of hundreds of SASP-related genes across large sample cohorts [41].

For the detection of soluble SASP components, the enzyme-linked immunosorbent assay (ELISA) represents a key analytical tool. This method enables both detection and quantitative measurement of SASP elements such as extracellular matrix remodeling enzymes, cytokines, growth factors, and chemokines, and remains the gold standard for identifying senescent cells, particularly in conditioned media and body fluids [40].

Biological age has traditionally been estimated using complex molecular markers derived from analyses of tissues and mixed cell populations, commonly referred to as biological aging clocks. However, emerging evidence emphasizes the importance of investigating aging processes at the single-cell level rather than at the level of the organism as a whole. Microglia, as the resident immune cells of the brain, exhibit distinct features during aging, and single-cell RNA sequencing (scRNA-seq) enables transcriptomic profiling of these cells across different life stages. To construct microglial aging clocks, scRNA-seq data are transformed into whole-transcriptome summaries or vector representations that can be used in machine learning (ML) models to predict cellular age. The traditional pseudobulk approach (PBHVG) sums the expression of the 3000 most variable genes within each cluster, whereas the Pseudobulk++ method integrates information on cell population frequencies and the expression of key genes into a single value representing a cluster. These approaches capture age-related changes in both gene expression and microglial cellular composition [42].

ML-based approaches are increasingly used to identify senescent cells, enabling cell classification based on morphological features as well as omics data, including transcriptomics, proteomics, and methylomics [43]. In the context of cellular aging, proteomics focuses on characterizing the SASP. Mass spectrometry-based proteomic technologies have substantially expanded the known composition of SASP and enabled the identification of factors involved in paracrine senescence, tumor invasiveness, inflammation, and hemostatic dysregulation. Quantitative analyses of secreted factors that mediate age-associated pathological phenotypes, as well as the identification of proteins that may serve as senescence biomarkers, make proteomics an important tool in aging research [44].

In one study, a comprehensive analysis of senescence was performed on postmortem brain tissue from donors with AD and neurologically healthy controls, using samples from the entorhinal, middle temporal, and somatosensory cortices. Immunostaining and multiple transcriptomic analyses, together with publicly available transcriptomic datasets, revealed increased senescence in the brains of individuals with AD compared with controls. The analyses also demonstrated an association between β-amyloid accumulation and microglial senescence, suggesting that this process is linked to reduced transcriptomic signatures related to phagocytosis and β-amyloid clearance [45].

RNA sequencing (RNA-Seq) is a transcriptome profiling method based on deep sequencing that has expanded understanding of the scope of eukaryotic transcriptomes and enables more accurate measurement of transcript levels and isoforms compared with other gene expression analysis methods [46]. In one study, RNA-seq was used to analyze transcriptomic changes during RS of human fibroblasts, allowing comparison of mRNA expression levels between young, middle-aged, and old cells across five different fibroblast strains. The sequencing results were validated by real-time PCR, confirming concordant directions of gene expression changes. RNA-seq also enabled comparison of differentially expressed genes among fibroblast strains, facilitating the identification of both shared and strain-specific transcriptomic alterations associated with cellular senescence [47].

Deep learning models have also been shown to predict cellular senescence based on nuclear morphology. Senescence predictions were associated with SA-β-gal activity, p16^INK4a^, p21^Cip1^, p53, and DNA damage markers. In a study using three fibroblast cell lines induced to senesce by ionizing radiation (IR) or RS, both conditions exhibited reduced DAPI staining intensity, consistent with previous observations. High-throughput microscopy images of DAPI-stained nuclei were analyzed using a U-Net neural network, and cell cycle analyses confirmed IR dose-dependent accumulation of cells in the G2 phase, indicating senescence-associated cell cycle arrest. These findings suggest that simple nuclear morphology parameters can serve as quantitative tools for assessing cellular senescence in vitro [48].

Other deep learning approaches, such as convolutional neural networks (CNNs), have significantly improved image classification accuracy and can be applied to identify senescent cells. In one study, senescence in human endothelial cells was induced using stressors such as hydrogen peroxide and camptothecin, and confirmed by SA-β-gal activity and increased p21 expression. CNNs demonstrated high classification accuracy, outperforming traditional ML methods, indicating their suitability for high-precision identification of senescent cells [49]. Additionally, the Cascade R-CNN system has shown promising performance in detecting senescent cells based on bright-field microscopy images. Using datasets annotated according to SA-β-gal activity and morphology, this approach effectively distinguished senescent from non-senescent cells and demonstrated high accuracy and reproducibility in identifying both replicative and doxorubicin-induced senescence [50].

The advent of single-cell RNA sequencing (scRNA-seq) has fundamentally expanded the capacity to resolve senescence heterogeneity within the central nervous system by enabling transcriptomic profiling at the level of individual cells rather than mixed tissue populations. This approach has been instrumental in revealing previously unrecognized senescent subpopulations that remain undetectable in conventional bulk analyses. For instance, scRNA-seq has facilitated the identification of disease-associated microglia (DAM), a transcriptomically distinct microglial subset that combines features of pathological activation and senescence, and has enabled the detection of senescent oligodendrocyte precursor cells accumulating in the vicinity of β-amyloid plaques in AD [45,51]. Furthermore, the construction of microglial aging clocks based on scRNA-seq data, using approaches such as Pseudobulk++ that integrate gene expression profiles with cell population frequencies, demonstrates how single-cell technologies can capture age-related transcriptomic shifts and compositional changes simultaneously [42]. As these methods continue to mature, they are expected to enable the identification of novel CNS-specific senescence biomarkers and the characterization of senescent cell states that are unique to particular brain regions, disease stages, or neurodegenerative conditions.

In parallel, ML approaches offer complementary capabilities that extend beyond cell identification toward clinically relevant applications. Deep learning models, including CNNs trained on nuclear morphology features of DAPI-stained cells, have demonstrated high accuracy in predicting cellular senescence and correlate well with established markers such as SA-β-gal and p21 [48,49]. Similarly, object detection frameworks such as Cascade R-CNN have shown robust performance in distinguishing senescent from non-senescent cells in bright-field microscopy images across different senescence-inducing conditions [50]. Beyond in vitro cell classification, these computational tools hold substantial potential for future clinical translation, including the stratification of patients according to their senescence burden based on tissue or biofluid profiling, the prediction of individual responses to senolytic or senomorphic therapies, and the discovery of novel senescence-associated molecular signatures through integration with multi-omics datasets. Importantly, the systematic validation of both scRNA-seq-derived senescence signatures and ML-based detection models in human CNS tissues remains a critical priority for translating these technological advances into reliable diagnostic and therapeutic tools for neurodegenerative diseases [42,43].

## 5. Cellular Senescence and Neurodegenerative Pathologies: Inflammatory, Vascular, and Self-Amplifying Mechanisms

### 5.1. SASP as a Driver of Neuroinflammation and Cellular Dysfunction

Cellular senescence in the CNS may contribute to neurodegeneration, in part, through SASP, a stress-responsive program that converts intracellular damage signals into extracellular cytokines, chemokines, growth factors, proteases, and other reactive mediators. In AD, neurons exhibiting senescence-like phenotypes have been reported and are linked to worse pathological and functional features, supporting the possibility that senescence is not only a correlation of aging but participates in disease mechanisms [52].

Importantly, the distinction between correlation and causation requires careful consideration. While numerous studies demonstrate the accumulation of senescent cells in neurodegenerative brains, stronger support for a causal contribution derives from genetic and pharmacological clearance experiments. In transgenic mouse models of tauopathy and amyloidosis, selective elimination of p16^INK4a^-positive senescent cells attenuated tau pathology, reduced neuroinflammation, and improved cognitive performance. Similarly, senolytic treatment (e.g., dasatinib and quercetin) reduced amyloid burden and restored aspects of synaptic function when administered at early disease stages. These interventional studies provide experimental evidence that senescent cells are not merely bystanders but can actively modulate disease progression, at least in preclinical systems. However, such causal evidence remains largely limited to animal models, and direct proof in humans is still lacking [53,54,55].

SASP-associated signaling has been associated with increased amyloid-β (Aβ) accumulation and tau pathology, including tau hyperphosphorylation, which are central lesions of AD [56]. However, much of the evidence remains associative, and directionality may differ by model and disease stage. In addition, senescence-linked changes in microglia and oligodendrocytes may further exacerbate injury through pathways related to Aβ plaque burden, tau dysregulation, and myelin degradation, suggesting that senescence-related programs span multiple neural lineages in degenerative brain states [57].

A major amplifier of SASP-associated injury is chronic glial activation. During AD progression, microglia can shift from protective surveillance and clearance toward persistent expression of inflammatory mediators such as IL-1β, IL-6, and TNF-α. This inflammatory profile has been linked to reduced Aβ clearance and increasing Aβ burden, thereby sustaining neuroinflammation. Astrocytes can likewise become chronically reactive, adopting neurotoxic features, with reported effects including increased inflammatory mediator release, reduced glutamate uptake, synaptic loss, and cognitive impairment [58]. Although astrocyte reactivity and cellular senescence are distinct programs, they may overlap and reinforce each other under persistent inflammatory signaling. More broadly, neuroinflammation represents a conserved injury-response program across neurological disorders, and persistent inflammatory signaling can magnify tissue damage across diverse etiologies [59].

SASP-linked inflammation also converges with oxidative stress and mitochondrial dysfunction. Excess ROS can promote mitochondrial DNA mutations, damage respiratory complexes, alter membrane permeability, and disrupt Ca2+ homeostasis, which are mechanisms implicated across AD, PD, and amyotrophic lateral sclerosis [60]. Senescent cells show impaired mitophagy, altered mitochondrial dynamics (including increased fission), and proton leak, which together reduce ATP efficiency while increasing ROS production [61]. Age-related declines in antioxidant defenses (e.g., catalase and superoxide dismutase) further reduce buffering capacity and increase vulnerability to chronic oxidative injury [62]. Notably, ROS and senescence can form a bidirectional loop: mitochondrial stress can induce senescence-like programs, and senescence-associated mitochondrial remodeling can further elevate ROS. At the circuit level, misfolded protein aggregates can form toxic oligomers that promote inflammation and synapse loss, and senescence-associated signaling has been proposed to contribute to synaptic dysfunction alongside tau aggregation [56].

### 5.2. Impact of Cellular Senescence on Blood–Brain Barrier and Neurovascular Dysfunction

Beyond its role in neuroinflammation, cellular senescence also critically affects neurovascular integrity. It may influence neurodegeneration through the neurovascular unit (NVU)—the coupled system of brain endothelial cells, pericytes, astrocytes, and neurons that maintains blood–brain barrier (BBB) integrity and matches local perfusion to neuronal activity. Senescent-like transitions across NVU cell types are proposed to promote pro-inflammatory signaling, weaken BBB homeostatic support, and contribute to neurovascular uncoupling, thereby increasing susceptibility to downstream neuronal stress [63].

Pericyte dysfunction is a plausible accelerator. Pericyte degeneration can blunt capillary blood-flow responses to neuronal activity, reducing oxygen and nutrient delivery and increasing metabolic stress. Age-associated pericyte loss has also been linked to hypoxia and impaired clearance of potentially harmful factors, which may further increase vulnerability to CNS disorders [63].

Mechanistically, SASP-like inflammation provides a potential bridge from cellular senescence to BBB compromise. Senescent astrocytes are reported to express SASP-associated proteases and other inflammatory mediators that can shift the extracellular milieu toward barrier instability. Matrix metalloproteinases (notably MMP-2/9) can directly disrupt tight junction proteins and increase BBB permeability, offering a concrete molecular route by which a protease-rich environment could erode barrier integrity [64].

At the molecular level, senescence-associated endothelial dysfunction involves downregulation and mislocalization of key tight junction proteins, including claudin-5, occludin, and zonula occludens-1 (ZO-1). SASP-derived cytokines such as IL-1β and TNF-α activate NF-κB signaling in endothelial cells, promoting transcriptional repression of junctional proteins and increasing paracellular permeability. In parallel, oxidative stress and mitochondrial dysfunction in senescent endothelial cells enhance reactive oxygen species production, which destabilizes adherens junctions through VE-cadherin phosphorylation and cytoskeletal remodeling. These alterations compromise barrier selectivity and facilitate leukocyte transmigration [65,66,67].

In vivo studies further demonstrate that endothelial senescence is associated with reduced nitric oxide bioavailability, impaired neurovascular coupling, and decreased cerebral blood flow responses to neuronal activity. Senescence-associated eNOS dysfunction and increased endothelin-1 signaling contribute to vasoconstriction and hypoperfusion. Importantly, experimental clearance of senescent vascular cells in aged mice partially restores vascular reactivity and reduces BBB leakage, supporting a functional contribution of vascular senescence to neurovascular impairment rather than a purely descriptive association [68,69,70].

Once BBB integrity is reduced, infiltration of blood-borne components and immune cells can amplify neuroinflammation [57]. BBB dysfunction is increasingly discussed as an early biomarker across multiple cognitive-disorder contexts; however, direct evidence that BBB disruption alone is sufficient to produce cognitive decline remains limited. In many models, BBB failure may function as a permissive accelerant that interacts with other pathologies rather than acting as a single initiating cause [71].

Finally, because cerebrovascular dysfunction and reduced cerebral blood flow (CBF) can precede overt cognitive impairment—and in some frameworks may precede classical neuronal pathologies—NVU senescence is plausibly positioned upstream of, or parallel to, neuronal stress and protein aggregation in at least some disease trajectories [72,73].

### 5.3. Senescence-Induced Senescence: Self-Amplifying Cycles of Tissue Dysfunction

A defining feature of the SASP is its capacity to propagate senescence-related dysfunction. SASP factors can reinforce senescence in an autocrine manner and induce senescence paracrinely in neighboring cells, thereby spreading damage across cellular and tissue compartments, as demonstrated in tumor models [74]. This propagation can be framed as autocrine reinforcement, paracrine spread, and reduced clearance of dysfunctional cells.

In neurodegeneration, one working hypothesis proposes a self-reinforcing loop in which tau activation promotes cellular senescence, while SASP signaling facilitates tau propagation and neurotoxicity, generating positive feedback between tau pathology and senescence burden [75]. Even if directionality varies by context, this feedback logic is consistent with progressive escalation of inflammation and proteostasis failure over time.

Microglial aging following stroke may sustain these dynamics at the tissue level, as impaired resolution of inflammation maintains elevated basal cytokine expression. IL-1α- and IL-6-associated signaling cascades can promote secondary senescence and drive astrocytes toward neurotoxic phenotypes [76]. Similar inflammatory and senescence-amplifying interactions are likely to operate in chronic neurodegenerative conditions.

Over time, the progressive accumulation of senescent cells across neural and vascular compartments, together with immunosenescence, is proposed to link vascular dysfunction with neural degeneration, thereby accelerating neurodegenerative progression [77,78].

### 5.4. Mechanisms Linking Cellular Senescence to Neurodegeneration in the CNS

Cellular senescence contributes to neurodegeneration through multiple, interrelated pathways that result in neuronal dysfunction, glia-mediated neuroinflammation, neurovascular compromise, and self-propagating paracrine signaling. In neurons, accumulated DNA damage and mitochondrial reactive oxygen species (ROS) contribute to senescence-associated functional decline, while the senescence-associated secretory phenotype (SASP) disrupts synaptic integrity, intracellular communication, and metabolic balance [25,26,27,32,33]. Senescent astrocytes and microglia adopt a proinflammatory secretome that fosters chronic cytokine release, perturbs glutamate homeostasis, diminishes phagocytic clearance of aggregated proteins, and thereby promotes a neurotoxic milieu [34,35,45,58].

Senescence also undermines neurovascular unit function; SASP factors with inflammatory cytokines and proteases compromise blood–brain barrier (BBB) integrity and impair neurovascular coupling, thereby facilitating immune infiltration and amplifying CNS stress responses [63,64,71]. Furthermore, the SASP propagates senescence in an autocrine and paracrine manner, reinforcing a cascade of inflammatory signaling and interacting with amyloid-β (Aβ) and tau pathologies to sustain degenerative feedback loops [56,74,75,76].

These mechanisms are not isolated but instead reinforce one another. Mitochondrial ROS serves both as an upstream inducer of senescence and as a downstream consequence of senescent cell activity; compromised BBB function augments inflammatory signaling that accelerates glial senescence; and impaired microglial clearance exacerbates pathogenic protein aggregation and tissue injury [57,60,61,71]. Therefore, cellular senescence functions as a system-level mechanism that links stress responses to chronic neuroinflammation, synaptic dysfunction, vascular instability, and the progressive nature of neurodegenerative disorders [20,24,77,78].

Taken together, these findings indicate that cellular senescence in the CNS is not a uniform process but rather exhibits marked cell-type and disease-specific heterogeneity, which carries direct implications for therapeutic targeting. In AD, senescent astrocytes and microglia emerge as predominant pathogenic populations, as they sustain chronic neuroinflammation, impair phagocytic clearance of β-amyloid, and promote synaptic destabilization through SASP-mediated signaling [45,79]. In PD, senescence predominantly affects dopaminergic neurons and astrocytes within the substantia nigra, where it facilitates α-synuclein aggregation and propagation, exacerbates oxidative stress, and amplifies neuroinflammatory cascades that drive progressive dopaminergic cell loss [80,81]. In MS, OPCs in demyelinating lesions represent the key senescent population, as their impaired differentiation capacity and SASP-mediated inhibition of remyelination contribute to the consolidation of demyelination and the transition to progressive disability [12,82]. This disease-specific distribution of senescent cell populations suggests that effective senescence-targeted therapies cannot rely on a universal approach but must instead be tailored to the predominant senescent cell types and their pathogenic roles within each neurodegenerative context. Consequently, the development of cell-type-selective senolytic or senomorphic strategies, capable of preferentially targeting senescent astrocytes and microglia in AD, dopaminergic neurons and astrocytes in PD, or senescent OPCs in MS, represents a critical priority for translating senescence research into disease-modifying interventions [56,83].

## 6. Alzheimer’s Disease

### 6.1. The Link Between Cellular Senescence and AD

Preclinical and human tissue evidence strongly links cellular senescence to AD pathology, and initial small clinical trials with senolytics show safety and CNS penetration, but clinical efficacy has yet to be proven. In the brains of AD patients, senescence of neurons, astrocytes, microglia, endothelial cells, and oligodendrocyte progenitors is observed, with increased expression of p16/p21, SA-β-gal, and SASP (IL-6, IL-8, MMP-3, etc.) [79,84,85]. AD neurons and directly induced patient neurons exhibit a stable ‘neuronal senescence state’ and pro-inflammatory SASP capable of inducing astrogliosis [86]. Near Aβ plaques in human and mouse brains, OPCs show particularly strong senescence; simply exposing them to Aβ in vitro is sufficient to induce this phenotype [51,56]. In many models (Aβ, tau, 3xTg), pharmacological or genetic removal of senescent cells reduces Aβ and tau burden, neuroinflammation, and improves memory, especially with early intervention [51,84,87]. Although the accumulation of senescent cells strongly correlates with amyloid and tau pathology, causal inference in humans remains limited. Importantly, interventional studies in transgenic AD mouse models demonstrate that genetic or pharmacological clearance of senescent cells reduces amyloid and tau burden and improves cognitive performance, suggesting a disease-modifying role. Nevertheless, whether senescence initiates pathology or amplifies pre-existing neurodegenerative cascades likely depends on disease stage and cellular context [53,79,88].

### 6.2. Senolytic Interventions

The most advanced strategy is intermittent administration of dasatinib + quercetin (D + Q). A pilot, open-label phase I study conducted in five patients with mild AD found good tolerance of the therapy, no significant changes in cognitive function over 12 weeks, and detected the presence of dasatinib in cerebrospinal fluid and changes in inflammatory biomarkers and selected senescence mediators [89,90]. The SToMP-AD study (NCT04063124) is a vanguard study preparing for a randomized phase II trial, focusing on CNS penetration, safety and SASP biomarkers [91]. Further studies using D + Q in patients with MCI or AD are ongoing to evaluate senolytic activity and observe the reappearance of senescent cells within 12 months [32,85,92]. However, literature reviews highlight significant challenges, such as the lack of standardized senescence biomarkers, the risk of toxicity (e.g., navitoclax showing non-selective neurotoxicity in human neuron models) and the need for early therapeutic intervention [32,87].

## 7. Parkinson’s Disease

### 7.1. Evidence for Senescence in PD

PD is typically associated with ageing, and many ageing processes (mitochondrial dysfunction, oxidative stress, inflammation, loss of proteostasis) overlap with the mechanisms of PD pathogenesis and promote the development of a cellular senescence phenotype [81,93,94]. A growing body of evidence indicates that not only neurons, but also glial and immune cells may acquire the characteristics of pathologically ageing cells in PD. Elevated expression of senescence markers (including p16^INK4a^, p21, SA-β-gal) and SASP factors (IL-6, IL-1α, IL-8, MMP-3) has been found in the brains of PD patients, particularly in astrocytes in the substantia nigra [81,95]. These markers are also increased in cerebrospinal fluid and the prefrontal cortex, along with signs of mitochondrial and telomere dysfunction [81,96]. In animal and cell models, overexpression of α-synuclein, its fibrils (PFFs), environmental toxins (paraquat, rotenone) and lipid overload induce a senescence phenotype in astrocytes, microglia and dopaminergic neurons, and the elimination of senescent cells alleviates dopaminergic damage [95,97,98].

### 7.2. Mechanisms Linking Senescence to Parkinson’s Disease Pathology

Senescence promotes chronic neuroinflammation through SASP, exacerbating oxidative stress and dopaminergic neuron damage [80,81]. α-Synuclein can itself induce DNA damage, p53 pathway activation, and senescence, and senescent neurons increase the secretion of α-synuclein aggregates, facilitating their spread between cells [97,99]. Mitochondrial dysfunction, defects in autophagy and the lysosomal system, and lipid accumulation (e.g., glucosylceramides in GBA disorders) further exacerbate oxidative stress and promote a senescent phenotype in SNpc (substantia nigra pars compacta) neurons [81,93,96]. It is therefore suggested that cellular senescence acts as an intermediary between the ageing process, genetic and environmental factors, and the degeneration of dopaminergic neurons [93,94,100].

## 8. Multiple Sclerosis

### 8.1. Current Evidence of Cellular Senescence in MS

Multiple sclerosis is increasingly recognised as a disease in which, in addition to classical autoimmune inflammation, processes associated with accelerated cellular ageing of the nervous and immune systems may occur. Cellular senescence has therefore been proposed as a potential contributor to neurodegeneration and the transition to progressive disability.

#### 8.1.1. Evidence from Human Studies

Post-mortem analyses of brain tissue from patients with progressive MS have demonstrated an increased number of cells expressing senescence markers (including p16, 53BP1 and lipofuscin) in both active and chronic demyelinating lesions, as well as in apparently normal white and grey matter. These senescent cells include neurons, astrocytes, oligodendrocytes, microglia and macrophages [12,96]. Importantly, a higher accumulation of senescent cells has been associated with faster disability progression and earlier mortality in MS patients [12]. Signs of senescence and immunosenescence have also been described in peripheral immune cells of MS patients, suggesting that ageing-related immune alterations may contribute to disease progression [101,102,103].

#### 8.1.2. Evidence from Experimental Models

Additional insights come from experimental systems. Senescence has been detected in SOX2-positive progenitor cells within demyelinating lesions and in neural progenitor cells (NPCs) and induced pluripotent stem cells (iPSCs) derived from patients with primary progressive MS [82]. Furthermore, studies in experimental autoimmune encephalomyelitis (EAE) and chemically induced demyelination models have demonstrated increased p16 and p21 expression in the brain and spinal cord, together with the accumulation of senescent microglia and macrophages [104,105]. Although these models provide mechanistic insight, their translational relevance to human MS remains limited and requires cautious interpretation.

### 8.2. Mechanisms Linking Senescence to Multiple Sclerosis Pathology

Several mechanisms have been proposed to explain how cellular senescence may contribute to MS pathology.

#### 8.2.1. Mechanisms Supported by Human Observations

Senescent glial and immune cells produce SASP, including cytokines (e.g., IL-6), matrix-remodelling enzymes (MMPs), chemokines and ROS. This pro-inflammatory secretome may sustain chronic low-grade inflammation behind the blood–brain barrier and potentially contribute to neurodegeneration in the progressive phase of MS [12,104,106].

#### 8.2.2. Mechanisms Proposed Mainly from Experimental Studies

**Scheme 1:**
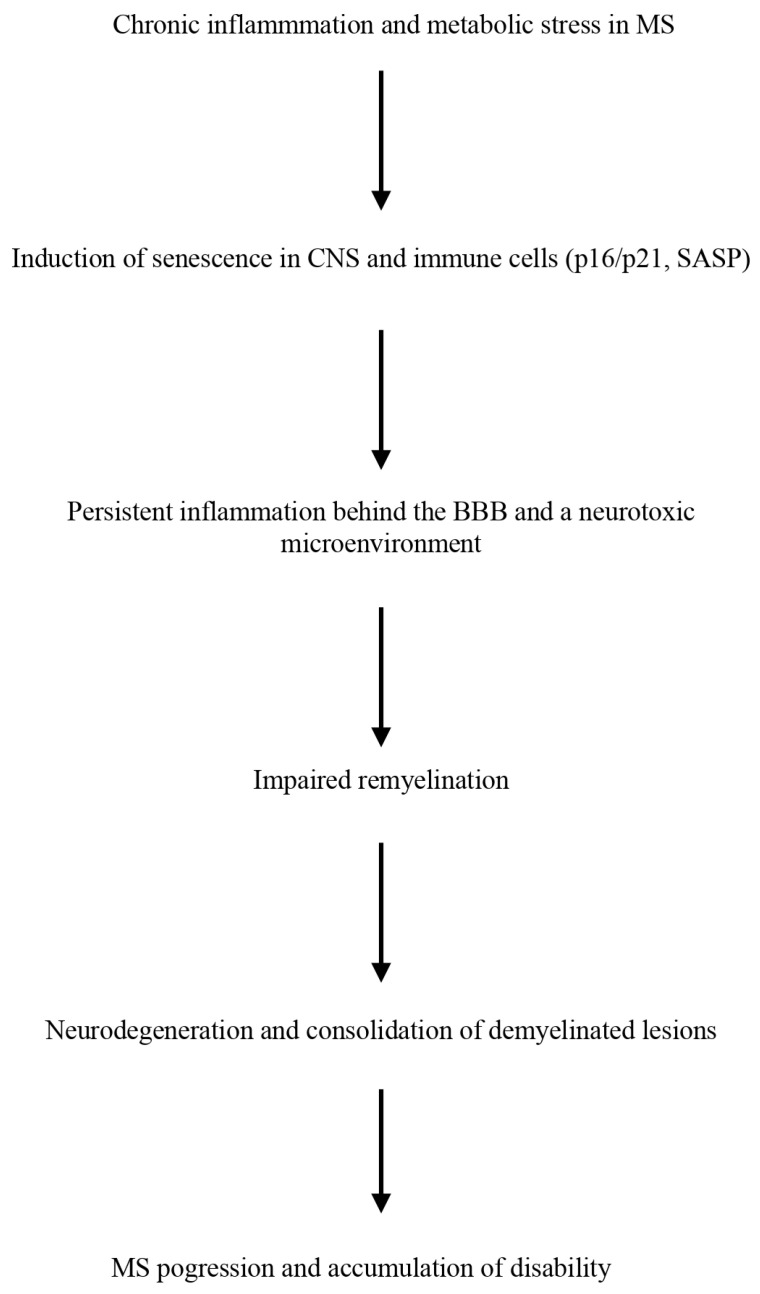
Senescence-linked cascade leading to progressive multiple sclerosis.

Experimental work suggests that senescence of oligodendrocytes and progenitor cells may impair remyelination. Factors such as HMGB1 and CCL11 (Eotaxin-1) released from senescent cells have been shown to inhibit oligodendrocyte progenitor cell maturation in experimental systems [82,107,108]. In addition, oxidative stress, mitochondrial dysfunction, iron and lipid accumulation, and chronic inflammasome activation—processes widely documented in MS pathology—are strong inducers of cellular senescence in experimental studies [96,104,109] (as shown in Scheme 1).

Experimental and translational studies also suggest that immunosenescence of B and T lymphocytes may alter immune responses, potentially affecting disease course and response to disease-modifying therapies (DMTs). However, the clinical significance of these observations remains incompletely understood [101,102,103].

### 8.3. Key Observations

Cellular senescence has been described in multiple CNS cell populations, including neurons, astrocytes, microglia and OPCs. Increased expression of senescence markers such as p16, p21, SA-β-gal and SASP factors (e.g., IL-6, IL-8 and MMP3) has been reported across several neurodegenerative disorders, including AD, PD and MS (as shown in Scheme 1).

In AD, senescence has been observed in neurons, astrocytes and microglia and is associated with β-amyloid and tau pathology. In PD, senescence is particularly prominent in astrocytes of the substantia nigra and in dopaminergic neurons. In MS, senescent cells appear to accumulate predominantly in oligodendrocyte progenitor cells and glial populations within demyelinating lesions [12,79,80].

The heterogeneity of senescence across different cell types and stress conditions has been demonstrated using multiple experimental approaches, including SA-β-gal staining, qRT-PCR, ELISA, single-cell RNA sequencing (scRNA-seq) and machine-learning-based analyses [37,40]. Nevertheless, most of these mechanistic insights derive from experimental studies, and further research is required to determine their clinical relevance in human neurodegenerative diseases. A concise summary of cellular senescence features across AD, PD and MS is presented in Table 3.

## 9. Therapeutic Strategies

An increasing body of experimental and clinical evidence indicates that cellular senescence represents a key pathogenic mechanism shared across neurodegenerative and neuroinflammatory disorders, including AD, PD, and MS. Cellular senescence, originally described as a tumor-suppressive mechanism, under conditions of chronic stress and organismal aging leads to the accumulation of cells that are permanently arrested in the cell cycle while remaining metabolically active and acquiring a characteristic secretory phenotype known as SASP [56,110]. Within the CNS, this phenomenon is of particular relevance, as the brain microenvironment is exceptionally sensitive to prolonged exposure to pro-inflammatory cytokines, chemokines, matrix metalloproteinases, and growth factors released by senescent cells.

In neurodegenerative diseases, senescence is not a uniform process but rather exhibits marked heterogeneity with respect to both its induction mechanisms and the cellular populations involved. In AD, the accumulation of β-amyloid and pathologically modified tau protein plays a central role by inducing chronic activation of the DDR, oxidative stress, and disturbances in proteostasis. These processes converge on the activation of the p53–p21 and p16^INK4a^–Rb signaling pathways, resulting in irreversible cell-cycle arrest and stabilization of the senescent phenotype [79,111]. Concurrently, mitochondrial dysfunction and excessive production of ROS amplify the SASP, creating a positive feedback loop between senescence and neuroinflammation that accelerates disease progression [79].

Within this context, glial cells emerge as the predominant senescent populations in the CNS. Senescent astrocytes lose their capacity to maintain neuronal ionic and metabolic homeostasis, exhibit impaired glutamate uptake, and promote oxidative stress. At the same time, they secrete high levels of pro-inflammatory mediators such as IL-6, IL-1β, and TNF-α, as well as extracellular matrix–modifying enzymes, which contribute to synaptic destabilization and BBB disruption [56,110]. Notably, senescent astrocytes frequently adopt a neurotoxic A1-like phenotype that actively participates in neuronal degeneration and exacerbation of amyloid pathology, rendering this cell population a particularly attractive therapeutic target.

In parallel, microglia—the principal innate immune cells of the brain—undergo profound functional alterations during neurodegenerative disease. Chronic microglial activation ultimately results in functional exhaustion and senescence, characterized by reduced phagocytic capacity, including impaired clearance of β-amyloid and myelin debris, alongside increased secretion of SASP-associated inflammatory mediators [83]. Of particular importance are disease-associated microglia (DAM), which combine features of pathological activation and senescence, thereby sustaining chronic inflammation and promoting neurodegeneration.

In MS, senescence-related mechanisms are largely centered on oligodendrocyte precursor cells. Senescent OPCs exhibit impaired proliferation and differentiation, leading to ineffective remyelination and persistent neurological deficits. Moreover, their SASP contributes to a localized pro-inflammatory milieu that further suppresses regenerative processes and promotes secondary senescence in neighboring cells [83]. These findings highlight senescent OPCs as a major barrier to successful myelin repair in MS, distinguishing its pathogenic mechanisms from those of AD and PD, where astrocytes and microglia play dominant roles.

Although neurons are postmitotic cells, multiple studies describe a phenomenon referred to as neuronal senescence or “amitosenescence”, characterized by activation of DNA damage signaling, increased p21 expression, and pronounced mitochondrial dysfunction. This phenotype correlates strongly with synaptic loss and tau pathology, suggesting that senescence-like changes in neurons actively contribute to neurodegeneration rather than representing a passive byproduct of disease [112]. In addition, senescence of cerebral endothelial cells has been implicated in BBB dysfunction, facilitating immune cell infiltration and exacerbating neuroinflammation [83]. Targeting endothelial senescence may represent a strategy to restore BBB integrity. Experimental models suggest that modulation of NF-κB signaling or reduction in oxidative stress in senescent endothelial cells improves tight junction organization and reduces vascular permeability, indicating that vascular-directed senotherapeutic approaches may exert indirect neuroprotective effects [113,114].

Recognition of the role of senescence in neurodegeneration has given rise to novel therapeutic concepts collectively referred to as senotherapy. Among the most intensively studied approaches are senolytics—agents that selectively eliminate senescent cells by targeting their senescence-associated anti-apoptotic pathways. In preclinical models of AD, PD, and MS, treatment with the dasatinib and quercetin combination has been shown to reduce the burden of senescent astrocytes, microglia, and OPCs, attenuate SASP signaling, and improve cognitive and regenerative outcomes [56,110]. Despite these promising findings, significant concerns remain regarding the long-term safety of senolytic interventions in the brain, particularly given the risk of eliminating cells that may retain compensatory or protective functions.

A key limitation of senolytic therapies is the potential risk of eliminating cells that may still perform physiological functions. Senescent cells are not always pathological; for example, they can participate in normal processes such as wound healing. Therefore, non-selective removal of senescent cells may lead to adverse effects. To address this issue, several strategies have been proposed to increase the selectivity and safety of senescence-targeting therapies. One approach involves the use of senomorphic compounds, which aim to attenuate the deleterious properties of senescent cells, including the SASP, without inducing cell death. Compounds such as rapamycin, JAK inhibitors, and metformin have been shown to modulate senescence-related pathways and reduce SASP-associated inflammation. In addition, drug delivery strategies based on prodrugs activated by senescence-associated β-galactosidase have been proposed to enhance selective targeting of senescent cells. Senolytic drugs may also be administered in intermittent dosing regimens, which limit excessive elimination of senescent cells while maintaining therapeutic effects. These approaches emphasize the importance of combining therapeutic efficacy with safety when developing senescence-targeted interventions [115].

Recent studies indicate that senomorphics, SASP modulators, and other innovative drug delivery systems, such as prodrugs activated by enzymes specific to senescent cells, enable more selective and safer therapeutic interventions. These strategies can be applied in intermittent regimens or combined with senolytics, thereby reducing excessive cell loss while preserving the beneficial physiological functions of senescent cells [116].

As an alternative to cell-eliminating strategies, senomorphics and SASP inhibitors aim to modulate the senescent phenotype without inducing cell death. Inhibition of key signaling pathways such as NF-κB, p38/MAPK, and mTOR has been shown to suppress SASP expression and reduce neuroinflammation. In this context, autophagy modulators, including rapamycin, have demonstrated the capacity to decrease senescence markers and improve neuronal and glial function in experimental models of AD [111]. These approaches are widely regarded as potentially safer for long-term treatment of chronic neurodegenerative conditions.

In addition, emerging immunotherapeutic strategies seek to harness the immune system to selectively recognize and eliminate senescent cells based on specific surface markers. Although still at an early experimental stage, such approaches represent a promising direction for future intervention [83]. Complementary supportive therapies targeting mitochondrial function, oxidative stress, and autophagic pathways may further limit senescence induction and mitigate its downstream effects [56,110].

Despite rapid advances in this field, all analyzed studies consistently emphasize substantial translational challenges. These include the lack of specific biomarkers for senescence in the human CNS, the difficulty of precisely targeting distinct senescent subpopulations such as A1 astrocytes, DAM, or senescent OPCs, and unresolved concerns regarding the long-term safety of senolytic therapies [56,83,110]. Consequently, senescence-targeted interventions remain largely confined to preclinical research, yet they represent a highly promising disease-modifying strategy for neurodegenerative disorders.

## 10. Discussion

### 10.1. Biological Mechanisms

Cellular senescence is induced by various stressors, including DNA damage, oxidative stress, and telomere shortening, leading to cell cycle arrest via the p53-p21 and p16^INK4a^-Rb pathways and the activation of the SASP regulated by DDR and epigenetic mechanisms [36]. Postmitotic neurons in the CNS exhibit features of senescence, such as DNA damage and SA-β-gal activity, while senescent glial cells (A1-like astrocytes, DAM) release cytokines, chemokines, and proteases, amplifying neuroinflammatory feedback and mitochondrial dysfunction [56]. In AD, β-amyloid induces senescence in OPCs, in PD, α-synuclein activates p53 and promotes protein aggregation, and in MS, SASP derived from OPCs inhibits remyelination through HMGB1 and CCL11 [51,82,97].

Cellular senescence is increasingly recognized as a feature of neurodegenerative disorders; however, the causal relationship between senescence and neurodegeneration remains incompletely understood [56]. While numerous experimental studies suggest that senescent cells may actively contribute to disease progression through SASP [56,117], alternative interpretations should also be considered. In certain contexts, cellular senescence may arise as a downstream consequence of ongoing neurodegenerative processes rather than acting as their primary driver [52,55,96]. Chronic neuroinflammation, oxidative stress, mitochondrial dysfunction, and the accumulation of pathological protein aggregates such as amyloid-β, tau, or α-synuclein can all induce cellular stress responses that promote the development of a senescent phenotype [7,81]. From this perspective, senescence may reflect a secondary response to neuronal injury and tissue damage [96,118]. It is therefore plausible that the relationship between senescence and neurodegeneration is bidirectional, forming a self-reinforcing cycle in which neurodegenerative pathology promotes senescence, while senescent cells further amplify inflammation, cellular dysfunction, and tissue damage [81,119]. Future studies, particularly in human tissues and clinical settings, will be essential to clarify the temporal sequence and mechanistic significance of cellular senescence in neurodegenerative diseases [7,118].

### 10.2. Clinical Relevance

The accumulation of senescent cells is associated with the severity of disease progression: in AD with amyloid accumulation and impaired phagocytosis, in PD with dopaminergic neuron degeneration, and in MS with disability progression and ineffective remyelination [12,45]. Elimination of senescent cells in experimental models (both genetically and pharmacologically) reduces amyloid/tau pathology, decreases neuroinflammatory responses, and improves cognitive function, particularly in the early stages of disease [51,80]. Senolytics, such as the combination of DQ, show penetration into the CNS and modify senescence markers in pilot AD studies [89]. These findings support a contributory and potentially causal role of senescent cells in disease amplification; however, current evidence does not conclusively demonstrate that senescence acts as a primary initiating event in human neurodegeneration [89].

### 10.3. Limitations of the Studies

The lack of specific biomarkers of senescence in the CNS increases the risk of false-positive results (e.g., SA-β-gal), and methods such as qRT-PCR or ELISA require the use of multiple markers for unambiguous identification [39]. Although correlations with pathology are strong, there is a lack of evidence of causality in humans. Clinical trials with DQ are still in their early stages and have not yet demonstrated cognitive improvement [89]. Additionally, senolytics carry the risk of neurotoxicity (e.g., navitoclax) and low selectivity, and their clinical application requires precise targeting of specific cell subpopulations [32].

An additional methodological challenge arises from the overlap between the senescence-associated secretory phenotype and general neuroinflammatory signaling [120]. Several SASP components, including cytokines such as IL-6 and IL-8, are also characteristic of activated microglia and reactive astrocytes [121]. Consequently, distinguishing a true senescent secretory phenotype from generalized chronic neuroinflammation in human CNS tissue may be technically difficult [122]. For this reason, the identification of cellular senescence typically requires the combined assessment of multiple senescence markers together with functional and morphological features rather than reliance on individual inflammatory mediators alone [123].

### 10.4. Directions for Further Research

It is necessary to accelerate phase II/III studies of senolytics (DQ, SToMPAD NCT04063124), taking into account SASP biomarkers and monitoring the re-accumulation of senescent cells [32]. It is also worth developing senomorphics, such as NF-κB, p38MAPK or mTOR (rapamycin) inhibitors, and immunotherapies targeting surface markers, which will allow safer modulation of senescence [83]. Standardisation of single-cell omics and ML methods will enable more accurate detection of heterogeneous senescence in human CNS tissues and evaluation of early interventions in MCI, prodromal PD and progressive MS [48].

## 11. Conclusions

Cellular senescence emerges as a unifying and biologically relevant mechanism underlying brain aging and the pathogenesis of major neurodegenerative diseases, including Alzheimer’s disease, Parkinson’s disease, and multiple sclerosis. Accumulating evidence indicates that senescent neurons, glial cells, vascular cells, and progenitor populations contribute to chronic neuroinflammation, impaired proteostasis, mitochondrial dysfunction, and progressive tissue damage through the senescence-associated secretory phenotype (SASP). Importantly, senescence in the central nervous system is highly heterogeneous and cell-type specific, with astrocytes, microglia, and oligodendrocyte precursor cells playing distinct but interconnected roles across different disease contexts.

The reviewed data suggest that cellular senescence may actively contribute to disease progression through self-amplifying inflammatory and degenerative feedback loops, although definitive evidence establishing senescence as a primary initiating cause in humans remains insufficient. Experimental studies consistently demonstrate that selective elimination or modulation of senescent cells reduces pathological protein accumulation, attenuates neuroinflammation, and improves functional outcomes, particularly when applied at early disease stages. However, translation of these findings to clinical practice remains limited by the lack of standardized, CNS-specific senescence biomarkers, incomplete understanding of causal relationships in humans, and safety concerns associated with senolytic therapies.

Overall, targeting cellular senescence represents a promising disease-modifying strategy for neurodegenerative disorders. Future progress will depend on improved single-cell resolution of senescence phenotypes, development of safer senomorphic and immunotherapeutic approaches, and well-designed clinical trials focusing on early intervention. A deeper understanding of senescence-driven mechanisms may ultimately enable more precise and effective therapies aimed at slowing or preventing neurodegeneration.

## Figures and Tables

**Table 1:** Key features of cellular senescence in major CNS cell types.

Cell Type	Triggers	Core Senescence Markers	SASP Profile/Functional Consequences	Pathological Relevance
Neurons (post-mitotic)	DNA damage, oxidative stress, mitochondrial dysfunction, protein aggregation	DNA DSBs, SA-β-gal activity, p21, mitochondrial ROS, GATA4 accumulation	NASP; secretion of chemokines (MCP-1, RANTES, MIP-2, GRO-1, MCP-3, EOTAXIN); altered electrophysiological signaling	Synaptic dysfunction; tau pathology; propagation of inflammatory signaling
Astrocytes	Chronic inflammation, oxidative stress, proteotoxic stress	p16, p21, SA-β-gal, lamin B1 loss, HMGB1 loss	IL-6, IL-1β, TNF-α, IL-8, NOS2; glutamate dysregulation; MMP secretion	Neurotoxicity; BBB instability; promotion of neurodegeneration and cognitive decline
Microglia	Aging, chronic immune activation, mitochondrial stress	p16, SA-β-gal, elevated ferritin	TNF-α, IL-6, IL-8 (NF-κB/p38-driven SASP); reduced phagocytosis	Sustained neuroinflammation; impaired clearance of protein aggregates
Oligodendrocyte lineage cells (OPCs)	Oxidative stress, inflammatory milieu	p16, p21, DNA damage markers	SASP-mediated inhibition of progenitor differentiation	Impaired remyelination; contribution to progressive MS
Endothelial cells (BBB)	Oxidative stress, vascular aging	p16, p21, SASP factors	MMP secretion; barrier destabilization	BBB dysfunction; neurovascular uncoupling

**Table 2:** Detection of cellular senescence in the brain.

Method	Markers/Features	Cell Type
SA-β-gal assay	SA-β-gal activity	General CNS cells
Cell-type-specific markers	p16, p21, HMGB1 loss, SASP	Astrocytes, microglia, neurons, OPCs, VSMCs, neuroblasts
qRT-PCR	SASP genes (IL-6, IL-8, chemokines, growth factors)	CNS cells in vitro/in vivo
ELISA	Soluble SASP factors	Conditioned media, body fluids
RNA-seq/scRNA-seq	Transcriptome, aging signatures	Microglia, fibroblasts

**Table 3:** Cellular senescence in selected neurodegenerative diseases.

Disease	Main Senescence Characteristics	References
Alzheimer’s disease (AD)	Senescence of neurons and glial cells; increased p16/p21, SA-β-gal and SASP; association with Aβ and tau pathology	[51,56,79,84,85,86,87,88]
Parkinson’s disease (PD)	Senescent astrocytes and dopaminergic neurons; SASP-driven neuroinflammation; α-synuclein-linked senescence	[80,81,93,94,95,96,97,98,99,100]
Multiple sclerosis (MS)	Senescent OPCs and glial cells; impaired remyelination; chronic inflammation in demyelinating lesions	[12,82,96,101,102,103,104,105,106,107,108,109]

## Data Availability

No new data were created or analyzed in this study.

## Abbreviations

The following abbreviations are used in this manuscript:

AD	Alzheimer’s disease
PD	Parkinson’s disease
MS	Multiple sclerosis
SASP	Senescence-associated secretory phenotype
CNS	Central nervous system
CDK	Cyclin-dependent kinase
ROS	Reactive oxygen species
DSBs	Double-strand breaks
SA-β-gal	Senescence-Associated Beta-Galactosidase
NASP	Neuronal Senescence-Associated Secretory Phenotype
DDR	DNA Damage response
IR	Ionizing radiation
RS	Replicative senescence
CNNs	Convolutional neural networks
ML	Machine learning
PBHVG	Traditional pseudobulk approach
qRT-PCR	Quantitative reverse transcription PCR
RNA-Seq	RNA sequencing
ELISA	Enzyme-linked immunosorbent assay
Aβ	Amyloid-β
NVU	Neurovascular unit
BBB	Blood–brain barrier
CBF	Cerebral blood flow
SNpc	Substantia nigra pars compacta
SOX2	SRY-Box Transcription Factor 2
DMT	Disease-Modifying Therapy
OPCs	Oligodendrocyte precursor cells
DAM	Disease-associated microglia
NPCs	Neural progenitor cells
iPSCs	Induced pluripotent stem cells
PFFs	Pre-formed fibrils
